# Comprehensive Approach to Analyzing Rare Genetic Variants

**DOI:** 10.1371/journal.pone.0013584

**Published:** 2010-11-03

**Authors:** Thomas J. Hoffmann, Nicholas J. Marini, John S. Witte

**Affiliations:** 1 Department of Epidemiology and Biostatistics and Institute of Human Genetics, University of California San Francisco, San Francisco, California, United States of America; 2 Department of Molecular and Cellular Biology, California Institute for Quantitative Biosciences, University of California, Berkeley, California, United States of America; University of Florida, United States of America

## Abstract

Recent findings suggest that rare variants play an important role in both monogenic and common diseases. Due to their rarity, however, it remains unclear how to appropriately analyze the association between such variants and disease. A common approach entails combining rare variants together based on *a priori* information and analyzing them as a single group. Here one must make some assumptions about what to aggregate. Instead, we propose two approaches to empirically determine the most efficient grouping of rare variants. The first considers multiple possible groupings using existing information. The second is an agnostic “step-up” approach that determines an optimal grouping of rare variants analytically and does not rely on prior information. To evaluate these approaches, we undertook a simulation study using sequence data from genes in the one-carbon folate metabolic pathway. Our results show that using prior information to group rare variants is advantageous only when information is quite accurate, but the step-up approach works well across a broad range of plausible scenarios. This agnostic approach allows one to efficiently analyze the association between rare variants and disease while avoiding assumptions required by other approaches for grouping such variants.

## Introduction

There is increasing evidence supporting the role of rare variants in both monogenic and complex diseases [1]–[6]. In parallel with this new sequencing technologies are providing an avenue for effective detection of rare variants in the human genome [7]. Such technologies are helping the 1000 Genomes Project catalogue less common variants (http://www.1000genomes.org). These advances in our ability to study rare variants should substantially improve our insight into the genetic basis of health and disease.

Evaluating the potential impact of rare variants on disease is complicated, however, by their uncommon nature. Several approaches have been proposed for the analysis of rare variants. On the one extreme is collecting such an enormous study sample that rare variants are detected sufficiently often to allow for testing each variant individually; for example, Nejentsev et al. [8] discovered a rare variant with minor allele frequency (MAF) 0.46% in Type I Diabetes cases and 0.67% in controls, using 17,730 individuals. Evaluating each individual rare variant will generally not be effective for smaller sample sizes or for variants that have even lower MAFs than that of Nejentsev et al. [8] due to data sparsity. In particular, conventional analyses may produce extremely unstable estimates of rare variant effects on disease and be essentially uninformative.

An alternative is to combine rare variants together into groups in a reasonable manner so they can be efficiently analyzed. Note that when we use “efficient” in this manuscript, we will always be referring to statistical power; computational time will be referred to as runtime. One might simply tabulate in cases and controls the number of individuals that have any rare variants (e.g., within a given locus), and contrast these counts. Morgenthaler et al. [9] have termed this the Cohort Allelic Sums Test (CAST). This approach essentially assumes that the rare variants have similar effects on disease. In other words, CAST gives equal weights to all rare variants combined together. It also treats individuals who are heterozygous and homozygous in an identical manner, although there will be few of the latter when studying rare variants.

Another option is to somehow weight each rare variant and then combine them. The optimal approach will upweight the variants most likely to cause disease and downweight variants that have no effect on disease. The weights could be calculated in a number of different ways. Madsen and Browning [10] propose weighting each allele by the inverse of the estimated standard deviation of the total number of mutations in the controls. Rare variants can also be simultaneously analyzed with common variants in a multivariate test, as in the Combined Multivariate and Collapsing (CMC) method [11]. Here, a multivariate test is constructed using a term for collapsed rare variants plus terms for each of the common alleles. This allows for collapsing variants only when needed due to their rarity, and analyzing more common variants on an individual basis.

The decision to aggregate rare variants – with or without explicit weighting – requires a number of strong assumptions about the similarity of their effects on disease. This raises a critical unanswered question: how to best combine rare variants for analysis? For instance, one might choose a minor allele frequency threshold to define what is “rare,” or choose a weighting scheme for the variants (even if constant weights). In addition, one might decide to only aggregate nonsynonymous variants in the coding regions [9] as these might be the most likely to cause disease [12]. Such a grouping could be further refined to only nonsynonymous variants that lead to putatively deleterious mutations that impair the function of the protein (e.g., using predictive algorithms such as SIFT [13], PMUT [14], or PolyPhen [15]). However, such algorithms vary in the information used, and can give different results, which would lead to different groupings of rare variants. For example, we found that the agreement among SIFT, PMUT, and PolyPhen in predicting the impact of mutations was only 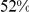 in the data we used for our simulation study (discussed below). Clearly it is very difficult to define *a priori* what rare variants should be aggregated into a single group for analysis.

Two methods have recently been proposed to collapse rare variants in a data-driven manner. Price et al. [16] extend the CAST [9] and the weighted approach [10] by testing multiple allele frequency thresholds, rather than choosing one fixed threshold, and also extend the test to quantitative traits. However, they assume that all rare variants are deleterious; while this may be a reasonable assumption for many diseases [2], there is also the possibility that some rare variants are protective. Han and Pan [17] allow for both deleterious and protective variants by letting the data determine whether an allele should be protective or harmful when collapsing, and also suggests collapsing common variants into the test. We combine and further extend these approaches in a more flexible data-driven model to decide how best to group rare variants for association analysis.

Our approach considers multiple possible groupings, choosing the “best” set based on statistical criteria, and correcting by permutation. One can use prior information from several sources to define these groupings; e.g., different protein coding function algorithms. Alternatively, or in addition, one can use data-driven methods to define these groupings based only on statistical criterion; e.g., all possible allele frequencies, all possible subsets of rare variants, or a “step-up” approach we propose here. That is, we use the data to decide whether a variant should be deleterious or protective, or whether the variant should even be in the model at all. We use a simulation study to evaluate these approaches. The simulations are based on data from deeply sequenced candidate genes in the one-carbon folate metabolic pathway [18].

## Methods

### General framework

Assume that we have undertaken a study of the relationship between 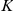 genetic variants and a phenotype 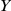 among 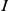 individuals. Let 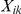 be the additive coding for a marker (i.e., the number of minor alleles individual 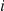 has at variant 
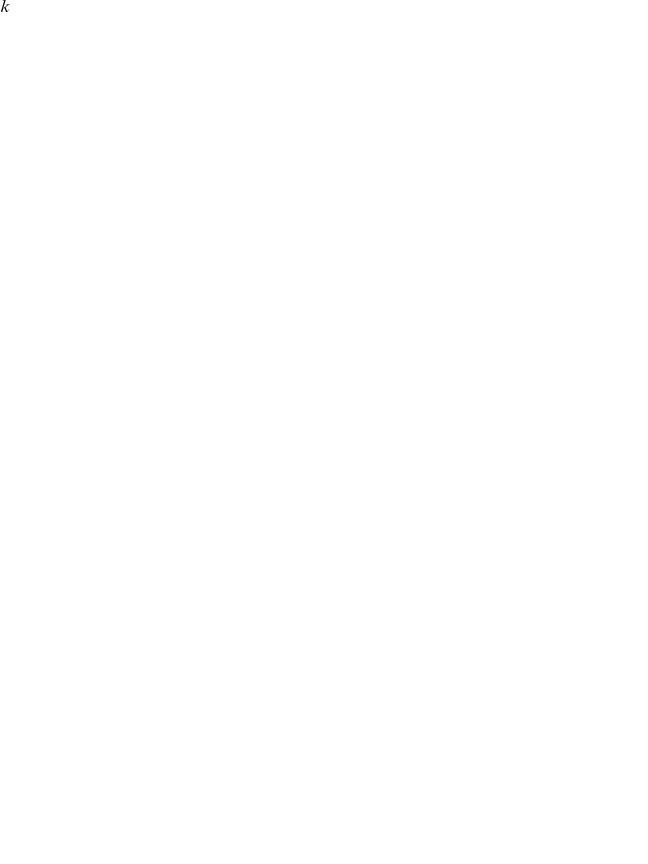
); others can be considered, but a dominant coding will be almost identical to an additive coding for a rare variant. Then a flexible disease model for the relationship can be given by
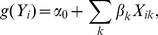
(1)where 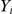 is an individuals phenotype (dichotomous or continuous) and 
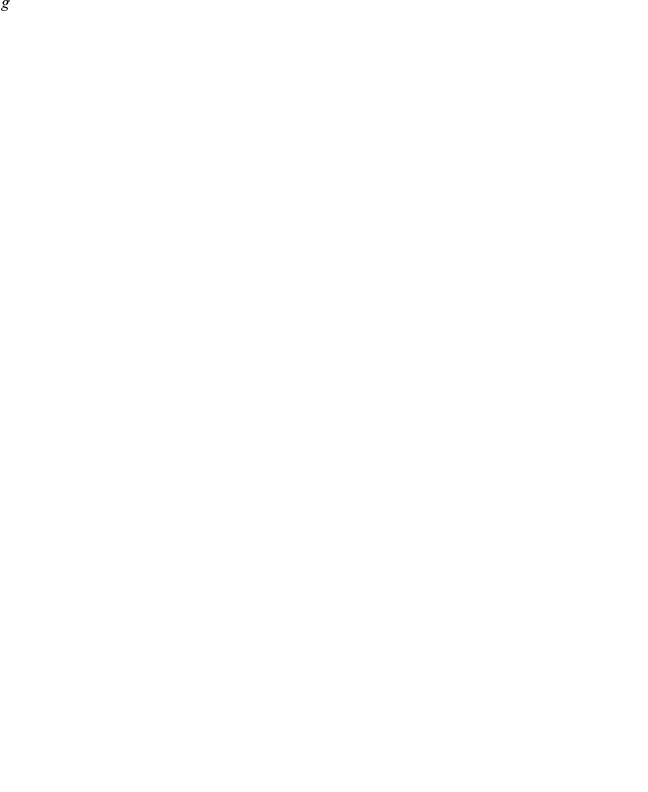
 is a link function (e.g., logit for logistic regression or the identity for linear regression). With rare variants, however, the data is too sparse to estimate each individual's 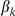. For example, suppose we try to fit a logistic regression to test for the genetic association of a rare variant with disease. Without an enormous sample size, the estimate of a single rare variant's effect on 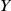 (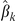) may be extremely unstable and essentially uninformative.

An alternative is to somehow aggregate multiple rare variants, and leverage their combined strength to improve estimation. This can be formalized with a second-stage model for the parameters of interest, a vector of coefficients 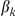


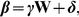
(2)where 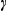 is a vector of combined genetic effects (e.g., a single collapsed effect, or two terms for a protective and deleterious effect) that we want to evaluate; 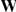 is a second-stage design matrix that incorporates information on factors about the genetic variants; and 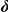 is a random effect. Equation 2 is essentially a prior model that distinguishes how one can “borrow information” across rare variants. Together equations 1 and 2 define a hierarchical model that can be used to incorporate complex interrelationships among the variants and their putative effects on disease.

However, most of the existing rare variant approaches essentially model a single combined genetic effect 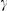, aggregating all of the data features into a single 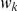 for each SNP, and assume 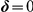. We build on these approaches, and for focus and tractability do not explore a fully parametrized hierarchical model; further details on the potential value of this approach are given in the discussion. Now combining Equations 1 and 2 gives the model
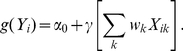
(3)That is, one is essentially modeling and estimating the effect of a weighted combination of variants 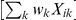.

We will explore different ways to model 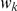 in this paper, from data-driven methods to those based completely on prior information. There have been several approaches proposed to modeling 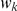 in the literature. The simplest is to set 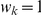 and sum them together. This is similar to the CAST approach [9], which uses an indicator variable for the presence of any rare variant. Here we use a multiplicative model 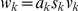, where 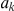 is a continuous weight (e.g., to incorporate allele frequencies), 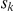 determines the direction of the variant effect (deleterious or protective), and 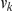 is an indicator variable determining whether the allele belongs in the model for variable selection. Note that in our description of these parameters below, we will be using the data to estimate them; we will correct for this by permutation at the end of the procedure.

For the continuous weight 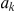, one can incorporate allele frequency information (or set this to 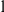). For example, Madsen and Browning [10] consider all alleles to be deleterious, and set 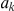 for dichotomous traits to the inverse square root of the expected variance based on allele frequencies 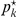 in the controls, 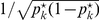, with pseudocounts (i.e., adding 1 to the numerator and denomerator when estimating 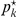 to prevent any zero weights). Price et al. [16] extend this to continuous traits by estimating the allele frequency 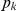 including all samples.

If we believe all variants have a deleterious effect, we can set 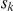 to be 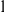, and ignore this parameter. Otherwise, we can let the data decide how to specify 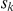. Han and Pan [17] addressed this first fitting a marginal regression model for the association between the variant and disease, and then flipping the coding of the genotype when the estimated coefficient is negative and reaches a certain significance threshold. We use a slightly different method for rare variants. For dichotomous traits, if an allele is more prevalent in controls than cases, we set 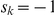 to indicate it is likely deleterious, and if it is more prevalent in cases than in controls, we set 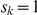 to indicate it is protective. For continuous traits we use the sign of the estimated covariance between the trait and marker; this is equivalent to the sign of the regression coefficient, just slightly faster to calculate.

Lastly, we have 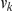, which determines whether a variable enters into the model. One example would be to set this by a hard minor allele frequency threshold (e.g., as in CAST [9]). However, we may also wish to try the approach at several allele frequency thresholds, or even all possible allele frequency thresholds [16]. In this case, we change our notation so that we are considering a set 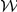 of models with elements indexed by a vector 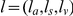 as 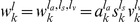. Testing all allele frequencies would be equivalent to running the test for each 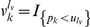, where 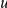 is the set of unique allele frequencies.

Another example of how to chose 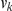 is as an indicator for variants in coding regions, since they may be more likely causal than those elsewhere [12]. We may wish to consider only those mutations that are nonsynonymous, and in particular those that are highly deleterious. Several algorithms exist for estimating the magnitude of the deleterious effect of mutations on protein function, but they do not always agree. Again, we might even also consider using several algorithms to define different groups to test. One may wish to use a consensus of all of these functional designations to group rare variants, or even use continuous information from the protein coding function algorithms. We can combine this with our ideas for testing multiple allele frequency thresholds.

There is one other model we will introduce for 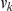, but it will be clearer after we describe the test statistic and understand its computational runtime. To speed up the approach one could use linear regression for all phenotypes, instead of logistic regression [16], [17]. We instead take the mean centered score of 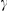 from Equation 3 divided by the empirical variance: 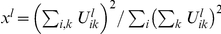, where 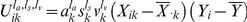, 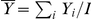, and 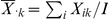. Then 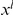 follows a chi-squared distribution with one degree of freedom. When we are considering a set of models 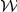 for 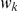, then the final test statistic of the procedure is given by 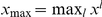. Then to compute the p-value of the test, we permute the phenotypes of the individuals, and recompute 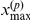 for permutation 
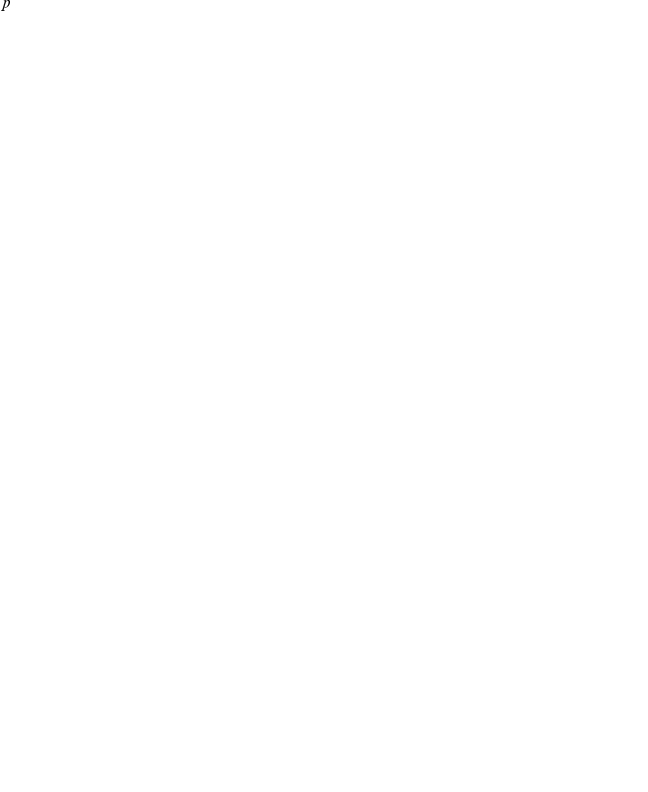
, following the entire procedure as before. Then the p-value for 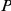 permutations is given by 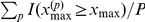.

With the computational complexity of testing multiple weights in mind, we also consider a data-driven method for specifying 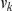. The approach we described above for testing all allele frequencies is computationally of order linear time in the number of variants. In contrast, having 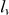 index all possible subsets of variants is on the order of factorial time in the number of variants, and is too computationally intensive for all but the smallest genes. Instead, we propose a “step-up” approach that has a computational runtime inbetween these two methods. This is similar to stepwise regression, but instead of selecting additional independent predictors, the step-up approach chooses the best combination of rare variants into a single aggregated group. With this approach we first compute the univariate test statistic 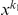 for each variant 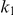. We then determine the “best” (i.e., 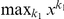) of these models; denote this model 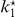, with test statistic 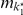. We then build on the model with variant 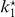 by computing the test statistic 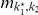 for each marker 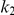 and the best marker 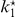 from the first approach. Denote the best added variant of this second step as 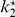. If 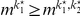, then the algorithm terminates. Otherwise, the algorithm continues until 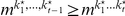. Again the p-value is obtained by permutation, repeating the entire procedure for each phenotype permutation. This algorithm's speed is of at worst a squared number of time in the number of variants.

We can further extend this to allow the set of all models considered to include any combination of the approaches from above, restricted to being computationally feasible. That is, 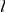 could index across all of the steps in the step-up model based on SIFT functional markers, and all of the steps in the step up model based on PMUT functional markers. This effectively uses the “best” of these two procedures. However, the more rare variant groupings and tests considered, the less efficient and more computationally intensive the approach will be compared to that which most accurately tests the true underlying model. When the disease model is not well understood, as is probably the case for many rare variants, it is advantageous to consider several different groupings and/or tests. In our simulations, we explore this trade-off between considering many possibilities and making strong assumptions.

### Models for variant weights

In the previous section we described a general framework and strategies for constructing a model for the variant weights 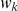 and evaluating an aggregated genetic effect on disease 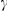. Here we enumerate the models that we will compare in our subsequent simulations (distinct from the models we will use to generate our data). We first investigated the following models with 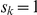 (i.e., all variants are deleterious) and 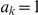 (i.e., they are equally deleterious):

1. *MAF* 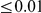: 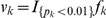, where 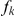 is defined:
  - *SIFT*: 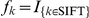 (this will be the true generating model, so as if we knew the true underlying model);
  - *Nonsynonymous*: 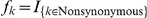 (modeling all mutations that alter protein coding function).
  This is similar to CAST, but summing 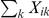 rather than an indicator variable of any mutation.
2. *MAF* 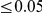: Same as (1), but 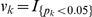.
3. *All MAF*: 
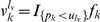
, where 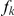 is (i.e., all allele frequencies as described above)
  - *Nonsynonymous*: 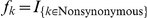;
  - *All protein coding*: 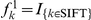, 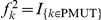, 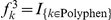 (i.e., try several protein coding functions since we will see they often differ);
  - *Non-generating protein coding*: 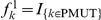, 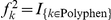 (i.e., exclude the protein coding function grouping information actually used to generate the data, and see if the other grouping methods, PMUT or polyphen, can still detect an association).
4. *Step*: 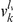 based on the “step-up” approach described above.

In addition to these, we then fit models 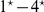, the same as 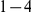 but with 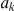 set to the inverse variance of variant 
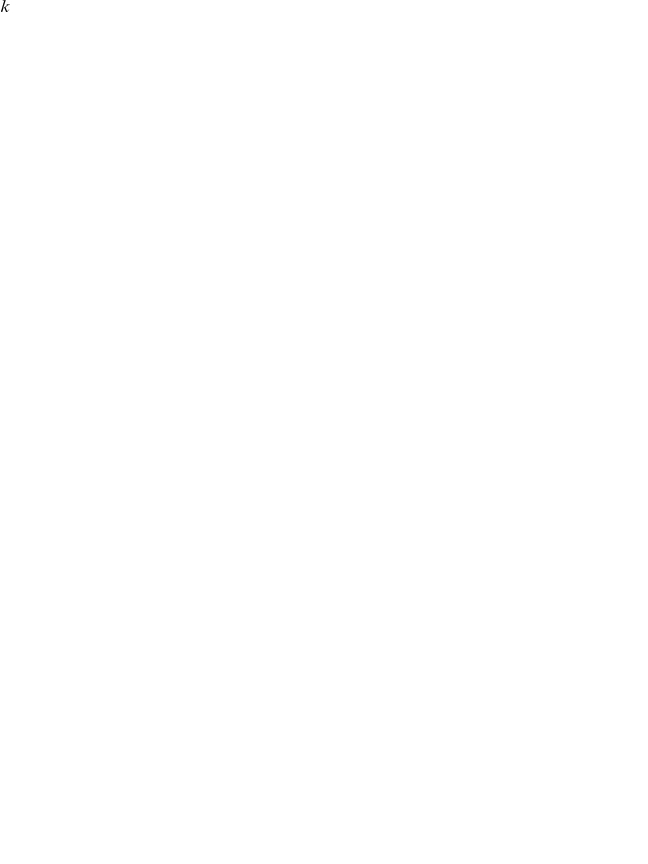
 using controls for dichotomous traits, and all subjects for continuous traits. Next we refit both models in 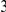 and 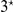, and choosing the “best”. Finally, we tested 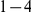 with 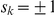 (i.e., signed, as described previously). Note that in these scenarios the weights presented here do not make as much sense for protective variants (i.e., especially weighting based on allele frequency in controls).

### Simulation design

We investigated several different rare variant disease models. Dichotomous traits were simulated using the disease model given in equation 1 under a logit link, and continuous traits with the identity link. We simulated a range of odds ratios (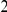 to 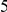) for dichotomous traits and mean differences (standard normal, 0.15 to 0.6) for continuous traits; a wide range of values are used here because rare variants are expected to have moderate to high penetrances [19], [20]. We also undertook simulations for an odds ratio of 1 or mean difference 0 to make sure the tests maintain the proper type I error. For dichotomous traits, 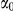 was chosen to keep the population prevalence fixed at 0.01. Other values for the population prevalence were considered, but did not materially affect the results. For continuous traits, 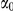 is irrelevant.

The variant data was generated using the haplotype frequencies across genes from an existing sequence-level dataset. One thousand cases were drawn according to the joint distribution of 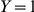 and 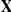, and 1000 controls from the joint distribution of 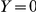 and 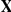, or 2000 individuals with a quantitative trait. A vector of genetic variants 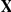 was drawn from haplotype frequencies of 480 individuals in which the coding regions of 16 genes in the folate metabolic pathway [18] were sequenced, in the California Newborn Screening Program; more results are given in the results section.

We ran 500 simulations per gene, and averaged the empirical power over all of the genes according to a type I error rate of 0.05 (i.e., average power for gene-specific detection, not pathway). We ran 500 permutations for each test (except CMC, for which an asymptotic test is available [11]). In practice one might wish to run a larger number of permutations for regions suggestive of association. 500 permutations were run here for simulation speed, as many tests were considered, and should be accurate for the simulations. Unless otherwise stated, we used the SIFT algorithm to determine if alleles were considered intolerant (including those with low confidence) and thus associated with disease, or tolerated and not associated with disease [13]. The power plots we present are the average over these genes. In each gene, we tried to construct and normalize our coefficients in such a way that the maximum contribution of any allele was less than or equal to the odds ratio.

We ran several simulations for dichotomous traits with the following values of 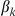 (Equation 1):

1. *Constant effect for all variants*: Let 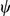 be the odds ratio, and 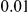 be the cutoff for whether an allele is rare and deleterious. Define 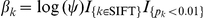.
2. *Varying the causal frequency*: Since we do not actually know the true allele frequency, we undertook several other simulations varying the “causal” rare allele frequency. That is, we allowed the cutoff 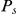 to follow a discrete uniform distribution according to the allele frequencies in each gene that were less than 0.05, varying this for each simulation. We define 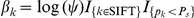.
3. *Continuous penetrance of disease*: Here, let 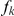 be the continuous coding of SIFT [13] for variant 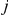, which ranges from 0 to 1, with 0 being predicted as more deleterious. We define 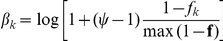. Variants that have a higher probability of deleteriousness as per the SIFT algorithm are simulated to increase the odds of disease proportionately higher.
4. *Incorporating rare and common variants*: We control how much more deleterious a rare variant is than more common variants with the parameter 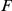 and define 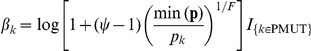. When 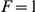, rarer variants have a very strong effect, and common variants have almost no effect. For larger values of 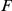, common variants have an increasing effect on disease. Note that here we use PMUT to increase the number of genes with deleterious common variants (four rather than one with SIFT).
5. *Incorporating protective and deleterious alleles*: We randomly partitioned each gene such that approximately 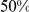 of the total allele frequency of rare functional variants were deleterious, and the rest protective. We define 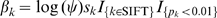, where 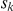 was 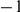 for deleterious alleles and 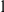 for protective alleles. We then repeated this with approximately 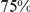 of the total allele frequency as deleterious.

We also reran simulations 1 and 5 for continuous traits. Here we replace the odds ratio 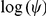 with the mean difference for each additional dosage of a variant allele, and sampling the trait according to a 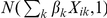 distribution.

## Results

### Dataset description

**Figure 1:**
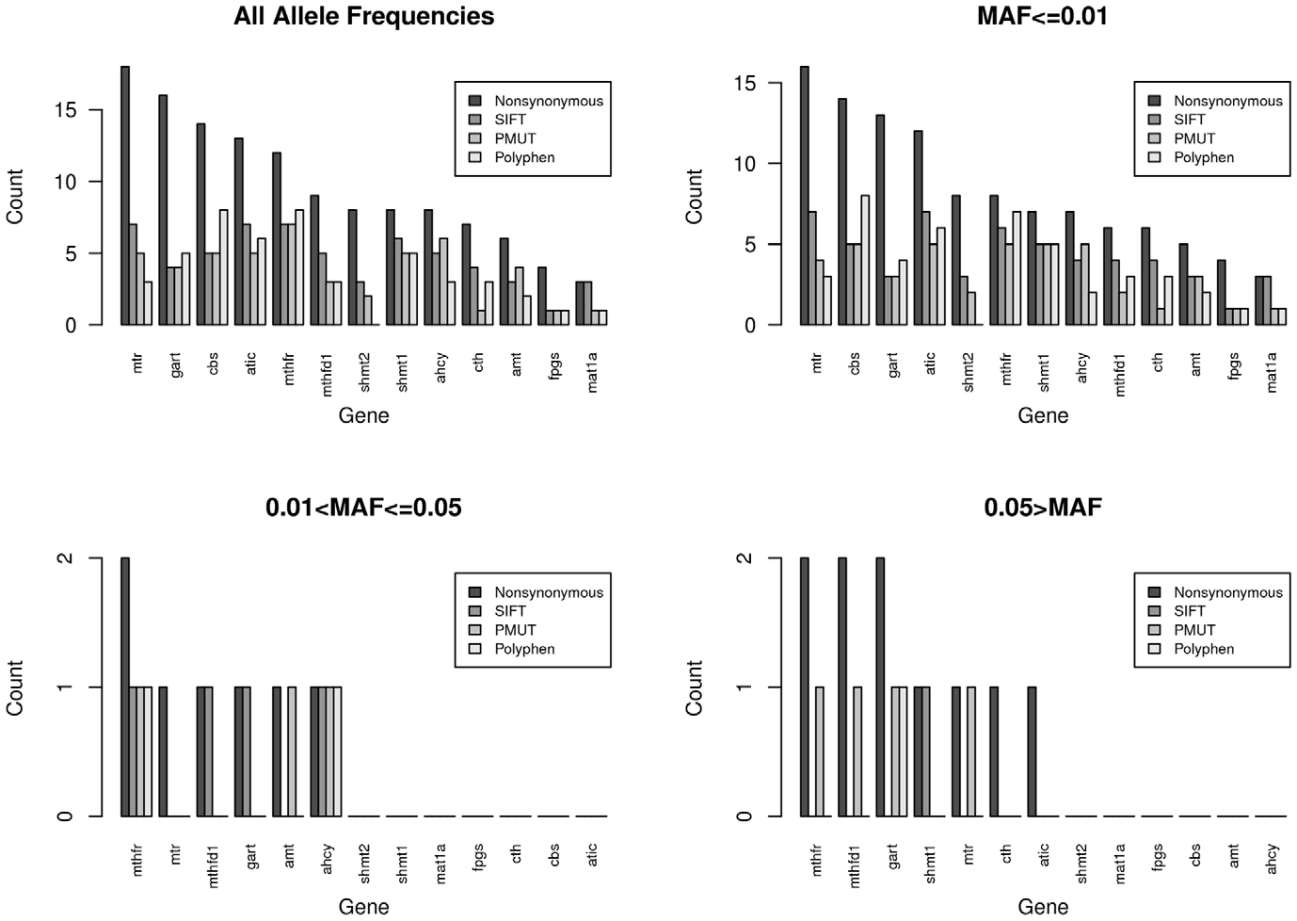
Deleteriousness of variants detected by sequencing one-carbon folate metabolic pathway candidate genes. For each gene, the number of variants from sequencing that are nonsynonymous, and then deemed deleterious by three different methods (SIFT [13], PMUT [14], or PolyPhen [15]) plotted by ranges of the variant's minor allele frequency. The SIFT designations are generally used here for our simulation studies (except those with common variants, where we used PMUT designations to have more genes with deleterious mutations for simulation purposes).

The deep sequenced dataset on which our simulations were based was rich with rare variants; out of 764 putative SNPs, 653 had allele frequencies less than 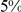, and 583 had an an allele frequency less than 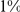. In the nonsynonymous regions of these genes we compared the SIFT [13], PMUT [14], and PolyPhen [15] methods of predicting whether the variants were deleterious protein coding mutations. Figure 1 shows the number of rare variants as characterized by these algorithms, for varying allele frequencies. We found that there was limited concordance among these methods (at best 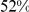, Table 1). This is similar to Chun et al. [21]. Nevertheless, the low concordance among these three algorithms is actually beneficial for our simulations because it adds variability reflecting reality. When we use SIFT to generate the disease model, it is interesting to assess how well the other approaches work. Data from 13 of the 16 genes were included in the analysis because each of the 13 had at least one intolerant nonsynonymous mutation as predicted by the SIFT algorithm (full details of this and other methods are in Table 1), whereas the remaining 3 had no predicted deleterious changes.

**Table 1:** Protein Function by Gene.

SIFT	PMUT	PolyPhen	Count
I	Path	Prob	8
I-LC	Path	Prob	2
I	Neut	Prob	**9**
I-LC	Neut	Prob	**1**
tolerated	Neut	Prob	1
I	Path	Poss	3
tolerated	Path	Poss	**1**
I	Neut	Poss	**6**
I-LC	Neut	Poss	2
tolerated	Neut	Poss	6
I	Path	Ben	**2**
I-LC	Path	Ben	**4**
tolerated	Path	Ben	**24**
I	Neut	Ben	**13**
I-LC	Neut	Ben	1
tolerated	Neut	Ben	43

Overlap of SIFT [13], T - Tolerated, I - Intolerant (tolerance index score 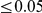, as suggested by the software documentation), I-LC - Intolerant with Low Confidence (tolerance index score 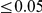, but median sequence conservation score 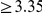); PMUT [14], Neut - Neutral, Path - Pathological; and PolyPhen [15], Ben - Benign, Poss = Possibly Damaging, Prob = Probably Damaging. Bolded counts indicate where one method is the opposite of the other, where we allow I-LC and Poss to go either way. There was a pairwise 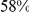 concordance between SIFT and PMUT, where we allowed SIFT I-LC to match to either PMUT pathological or PMUT neutral; 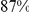 concordance between SIFT and PolyPhen, where we allowed SIFT I-LC to match to anything PolyPhen; and 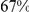 concordance between PMUT and PolyPhen where we allowed SIFT I-LC to match to anything and PolyPhen Poss to match to anything.

### Simulation results

**Figure 2:**
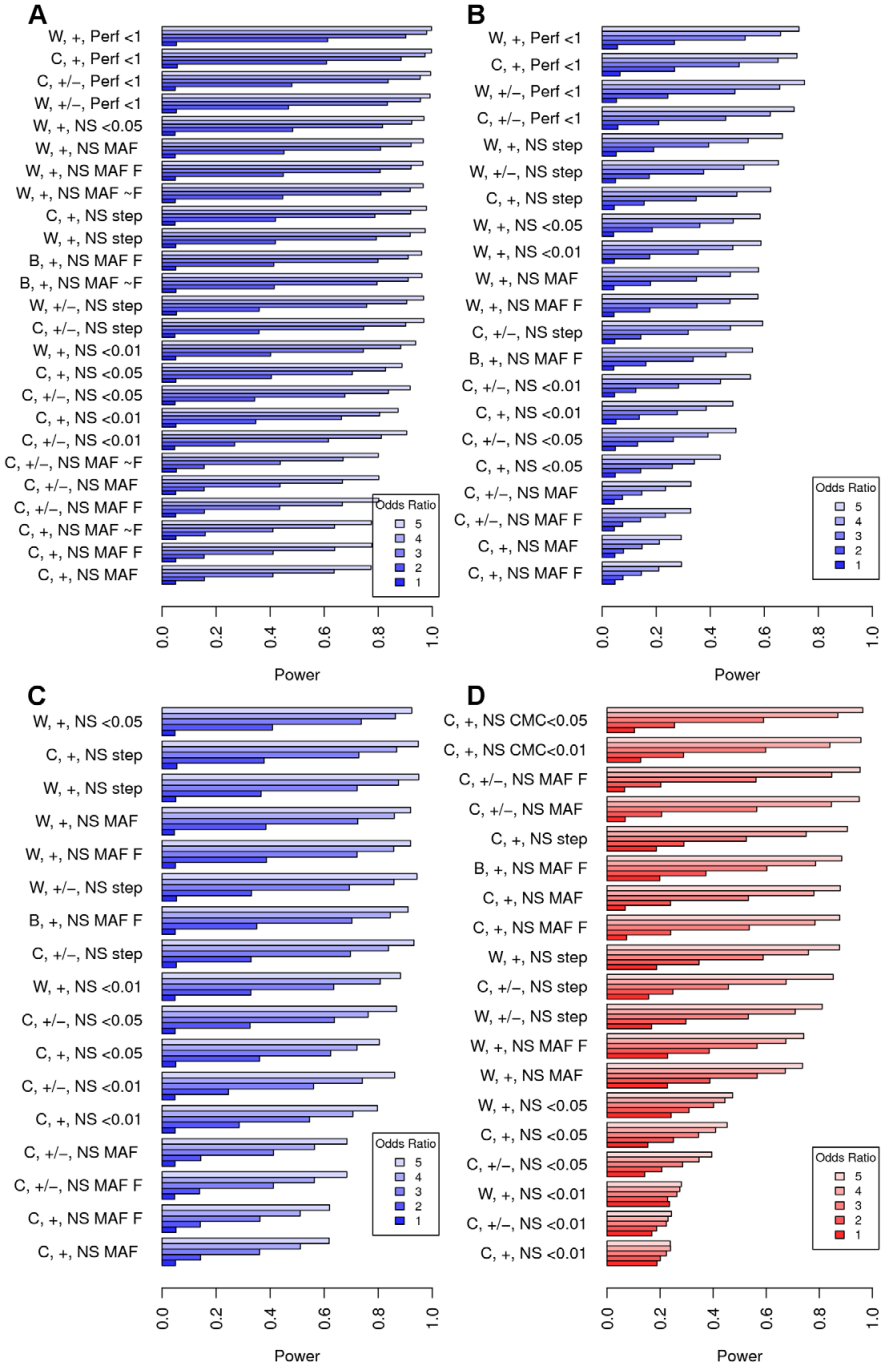
Results from simulation study comparing power for rare variant analysis approaches. 500 simulations were based on haplotype distribution for each of 13 deep sequenced candidate genes, and averaged. 500 permutations were run per test. Information for each situation on the bottom of each plot consists of three parts that indicate the test used: 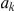 (‘C’ for constant, ‘W’ for weighted by allele frequency); 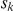 (‘
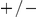
’ if signed, ‘
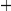
’ if constant); and the range of groupings 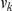 (‘NS’ for nonsynonymous, ‘F’ for all protein coding, ‘
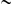
F’ for nongenerating protein coding, ‘MAF’ for all MAF, ‘step’ for step-up, and ‘Perf’ for the exact generating alleles when appropriate). Results in plots A-C are sorted by the plot that has the highest area, i.e., the most powerful overall. In D, each value of 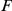 indicates how much common variants affect disease and must be considered separately; to emphasize this, we have sorted by the power when 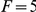.

**Figure 3:**
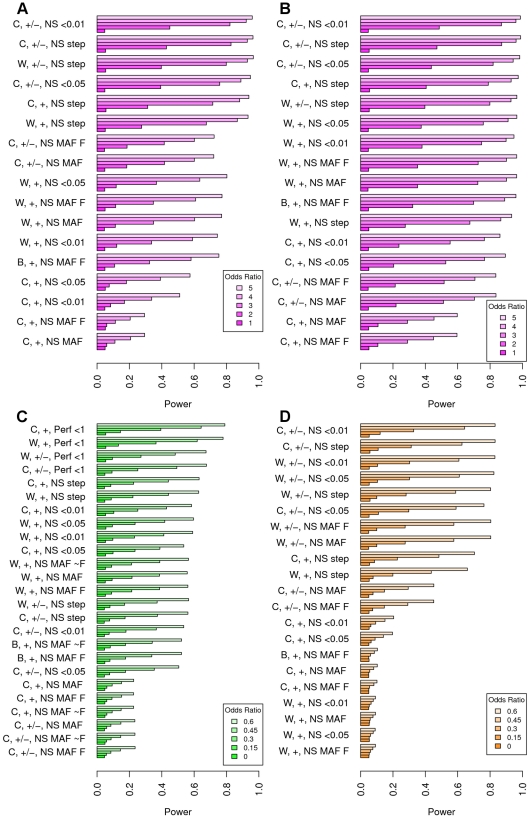
Further results comparing power across rare variant approaches. Results in Figures A and B show the effect of having both deleterious and protective rare variants. Figures C and D switches to a continuous trait, with Figure D showing the effect of having both deleterious and protective rare variants. Results are sorted by the plot that has the highest area, i.e., the most powerful overall. See the Figure 2 legend for additional details about the different simulations.

Each simulation enumerated above is highlighted in Figures 2 and 3. In these figures, the different scenarios are distinguished by the three indices separated by commas along the X-axes. The first label indicates which of the four tests was used (i.e., the model for 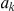): constant (C), weighted (W), or both constant and weighted (B). The second label is for the parameter 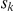 and indicates whether the sign was set to a constant 1 (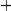), or allowed to vary as described above (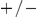). The third label is for the model parameter 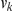, and indicates whether the test was done restricting to a particular algorithm's deleterious call (e.g., SIFT) or all nonsynonymous changes (NS), and what range of alleles or groupings that test was applied to. The latter corresponds to: the exact generating alleles (Perf for “perfect”, i.e., testing only the alleles contributing to disease), all allele frequencies (MAF), all functional groupings (F), all functional groupings except that used to generate the data (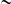
F), a hard allele frequency threshold (e.g., “
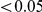
”), the CMC method with a hard threshold (only run for common variants, simulation 4), or the step-up algorithm described in the methods section (step). Unless otherwise stated, the order of the tests in the plots are by the most overall powerful (averaged over the 4 ORs or mean differences).

Figure 2A shows the results from simulation 1, the fixed MAF threshold of 0.01. The weighted method generally performs better than constant weights (even when we are testing the exact markers we use to generate, Perf) and appreciably better than applying constant weights to all minor allele frequencies as does using a fixed threshold (e.g., 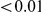 or 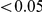). We also note that the step-up method also performs well in this circumstance. Lastly, signing the variants does not make the power much worse even though all SIFT variants are assumed deleterious. Figure 2B shows the results from simulation 2, under the more realistic scenario with different allele frequencies generating each simulation. Here the step-up method performs the best, aside from the unrealistic Perf test. In comparison with simulation 1, we see a more dramatic power reduction for the unweighted (C) tests that allow for multiple MAFs. Figure 2C shows the results from simulation 3, with a continuously generated deleteriousness of alleles. Surprisingly, the weighted method with a MAF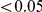 for aggregating variants has the most power in this figure. However, the step-up is nearly identical (C or W). As above, the weighting by minor allele frequencies in controls (W) generally worked better than not weighting (C). In these tests a similar step-down approach was tried, but it did not work well (results not shown).

We then looked at the effect of common variation according to the PMUT algorithm in simulation 4 (4 genes had common variants, Figure 1) [14]. In Figure 2D we vary the parameter 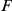 for each situation, and fix the odds ratio at 2. Here the order of the tests is not as informative as it was for the other plots; it is best to separately consider the different approaches' power for each value of 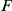 in Figure 2D. To emphasize this, Figure 2D is ordered by the power at 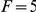. For 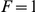 and 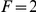, the rare variant methods perform the best. Step-up performs well, but we see a small power loss for the 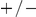 approach, unlike before. However, if common variants have any appreciable effect on disease (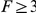), then the CMC approach works best. This is likely because it is more flexible and does not assume that the more common variants have the same effect at the expense of a few degrees of freedom. As expected, we also saw that requiring a hard cutoff of MAF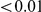 or 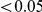 performed poorly (Figure 2D).

In the top panels of Figure 3 we can see the effect of protective and deleterious mutations (simulation 5). Figure 3A shows a 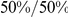 split, while 3B shows a 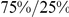 split of deleterious vs. protective variants. It is not surprising that the methods which sign variants based on case-control differences generally performed the best here, especially for the 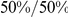 split. What is slightly surprising is that the unsigned step-up routine performs nearly as well as the signed step-up routine that does not. Even the constant threshold performs well, if it is signed. The unsigned methods look slightly better in the 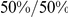 split than they do in the 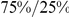 split, although the signed methods are preferred.

When considering continuous traits our simulations gave generally similar results as seen for dichotomous traits. Figure 3C shows results for simulation 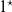 - data generated from SIFT prediction where all variants with MAF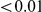 are causal. Results are similar to simulation 1 with the weighted and step-up approaches performing best, and allowing for any MAF doing worse. Figure 3D presents results for simulation 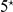 for the 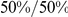 split. For continuous data, the signed tests show even more benefit than for dichotomous traits. In fact, assuming that all variants are deleterious works quite poorly, except for the step-up approach, which still did reasonably well.

## Discussion

We have compared several different approaches to rare variant analysis that incorporate varying amounts of prior information in deciding how to aggregate such variants. When one does not know how rare variants affect disease, and is hesitant to make the strong assumptions required to collapse them together, the completely agnostic step-up approach presented here may be the most appropriate. It performed either the best, or close to the best (excluding the “perfect” but unrealistic tests) in the various situations considered.

When it is possible that both protective and deleterious variants are present, we found it useful to sign variants (although little difference between stepwise and signed stepwise). Signing variants greatly improved the efficiency when both protective and deleterious variants are present, although some efficiency was lost when only deleterious alleles were present. The weighting schemes we considered based on allele frequency (models for 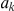) generally did not work well when both protective and deleterious variants were present. However, these weights were designed for the situation when all alleles are deleterious, and do improve the efficiency in those situations (with the exception of step-up, where there is little difference). Using a hard cutoff performed relatively poorly unless it accurately reflected the underlying disease model; aside from that, a slightly higher allele frequency threshold generally worked better. When using a slightly softer assumption of testing all MAF thresholds, we found that incorporating functional information from protein coding function algorithms generally improved the efficiency of the test, and added only a minor extra computational burden. Note, however, that we used the SIFT algorithm to generate this data in our simulations, so it is biased towards using that information. Yet even the other protein coding function algorithms (e.g., PMUT, PolyPhen) did well with all MAF when this information was not available. The more flexible step-up approach does not need to rely on having such information.

Our simulations focused on combining rare variants within particular genes. One can extend this approach to pathways, exomes, or entire genomes, although the latter may be computationally challenging. Some computational time may be saved by using an adaptive permutation that stops earlier for genes or regions that appear to have no impact. For exomes, one could also further collapse entire pathways instead of genes. A fast analysis of different pathways could be done by testing each gene individually, and combining the resulting p-values with the Fisher product test statistic [10], or applying another step-up approach to further combine the aggregated scores from each gene. Testing all MAF instead of the step-up approach is also an alternative if computational time is an issue [16].

Many complex diseases are likely due to a combination of rare and common variants. One can jointly analyze rare and common variants as in the CMC approach [11], but the rare variants must have a large enough effect size to contribute much to the efficiency of the test. Note that we did not consider various groupings for the CMC test because multivariate logistic regression was prohibitively slow for us to run many permutation tests in the simulations. An alternative may be using linear regression. In practice a combination of some of rare variant aggregation methods with the CMC method might be the most appropriate for many risk loci.

Another promising approach for rare variant analysis is hierarchical modeling [22]–[25]. We presented a general model in equations 1 and 2 that is essentially hierarchical, and even made some explicit prior assumptions about the variant effects distribution (e.g., a point mass with no variability). Further extending these models with other hyperparameters offers an opportunity to potentially improve upon existing rare variant techniques and is an important area of future research.

As with any genetic analysis, one may need to adjust for potential confounding (e.g., due to population stratification). Dichotomous covariates, or covariates with only a few levels, can be included easily in these rare variant approaches by stratifying on them. Otherwise the residuals of a logistic/linear repression of the trait on the covariates of interest can be fit with the continuous version of the test. One could also just use the model in Equation 1 adjusting for covariates; here, one might always use linear regression as it will be faster. The score test from linear regression is nearly the same as the score test from logistic regression, with the modification that the information contributions of each subject is weighted by 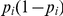, where 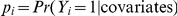, rather than an assumed constant residual variance as in ordinary linear regression.

In summary our simulations suggest that the step-up approach works quite well without requiring *a priori* information about how to aggregate rare variants for analysis. This agnostic approach was generally one of the best under a broad range of scenarios, and should perform well under disease models different than those considered here. Of course, when one knows the underlying disease model, aggregating rare variants to reflect this information will excel. In practice, however, combining rare variants may require strong and sometimes conflicting assumptions; softening such assumptions with a hierarchical model may prove valuable for rare variant analyses. Software for the approaches considered here is freely available in the R package “thgenetics” available from CRAN (http://cran.r-project.org/).
